# Identification, Baculoviral Expression, and Biochemical Characterization of a Novel Cholinesterase of *Amblyomma americanum* (Acari: Ixodidae)

**DOI:** 10.3390/ijms24097681

**Published:** 2023-04-22

**Authors:** Kevin B. Temeyer, Kristie G. Schlechte, Aaron D. Gross, Kimberly H. Lohmeyer

**Affiliations:** 1 Knipling-Bushland U.S. Livestock Insects Research Laboratory and Arthropod Genomics Center, USDA-ARS, 2700 Fredericksburg Road, Kerrville, TX 78028, USA; 2 Molecular Physiology and Toxicology Laboratory, Department of Entomology (MC 0390), Virginia Polytechnic Institute and State University, Latham Hall (Rm 307), 220 Ag Quad Lane, Blacksburg, VA 24061, USA

**Keywords:** salivary cholinesterase, synganglion, ixodid tick, lone star tick, vector-borne disease

## Abstract

A cDNA encoding a novel cholinesterase (ChE, EC 3.1.1.8) from the larvae of *Amblyomma americanum* (Linnaeus) was identified, sequenced, and expressed in *Sf21* insect cell culture using the baculoviral expression vector pBlueBac4.5/V5-His. The open reading frame (1746 nucleotides) of the cDNA encoded 581 amino acids beginning with the initiation codon. Identical cDNA sequences were amplified from the total RNA of adult tick synganglion and salivary gland, strongly suggesting expression in both tick synganglion and saliva. The recombinant enzyme (rAaChE1) was highly sensitive to eserine and BW284c51, relatively insensitive to tetraisopropyl pyrophosphoramide (iso-OMPA) and ethopropazine, and hydrolyzed butyrylthiocholine (BuTCh) 5.7 times as fast as acetylthiocholine (ATCh) at 120 µM, with calculated *K*_M_ values for acetylthiocholine (ATCh) and butyrylthiocholine of 6.39 µM and 14.18 µM, respectively. The recombinant enzyme was highly sensitive to inhibition by malaoxon, paraoxon, and coroxon in either substrate. Western blots using polyclonal rabbit antibody produced by immunization with a peptide specific for rAaChE1 exhibited reactivity in salivary and synganglial extract blots, indicating the presence of AaChE1 antigenic protein. Total cholinesterase activities of synganglial or salivary gland extracts from adult ticks exhibited biochemical properties very different from the expressed rAaACh1 enzyme, evidencing the substantial presence of additional cholinesterase activities in tick synganglion and saliva. The biological function of AaChE1 remains to be elucidated, but its presence in tick saliva is suggestive of functions in hydrolysis of cholinergic substrates present in the large blood mean and potential involvement in the modulation of host immune responses to tick feeding and introduced pathogens.

## 1. Introduction

The lone star tick, *Amblyomma americanum* (Linnaeus), is widely distributed across the Eastern United States; it is aggressive and non-specific when seeking hosts [1] and is known to vector pathogens causing diseases in animals and humans [2]. Recent modeling of the potential effects of climate change suggests that *A. americanum* could expand its range in North America and potentially become established in parts of Europe and Asia [3,4]. In addition to the potential threat of vector-borne disease, lone star tick bites to humans can cause the development of alpha-gal meat allergy, manifested as potentially life-threatening anaphylactic allergic reactions after consumption of non-primate mammalian meat or meat products [5,6,7]. Alpha-gal meat allergy is triggered by the oligosaccharide galactose-alpha-1,3-galactose (alpha-gal), absent in humans and other primates, but present in non-primate mammalian meat and in the saliva of *Amblyomma americanum* [5,8].

We recently reported the presence of acetylcholinesterase (AChE) in the saliva of ticks and other arthropod vectors and proposed that arthropod cholinesterase may function at the parasite–host interface to hydrolyze acetylcholine in host tissue at the parasite bite site, modulate subsequent host immune responses to promote successful parasite feeding, and possibly incidentally facilitate the establishment of pathogens transmitted in the tick saliva [9,10,11]. Although acetylcholine is best known as a neurotransmitter, it has been found to exert diverse and important signaling and regulatory functions [12]. Non-neuronal acetylcholine is secreted and maintained by tissues and cholinergic signaling participates in the control of many cellular activities, including immune function and homeostasis [13]. We previously expressed and biochemically characterized recombinant AChEs from the cattle tick *Rhipicephalus (Boophilus) microplus* and several other blood-feeding arthropods [11,12,13,14,15]. Our previous work reported that cholinesterases were present in the saliva of blood-feeding arthropod vectors but largely absent in the saliva of non-vector blood-feeding arthropods [11]. Here we report the discovery, baculoviral expression, and biochemical characterization of a novel recombinant cholinesterase (ChE), rAaChE1, from *A. americanum*. We further present evidence of AaChE1 in tick saliva and synganglia and discuss its potential functional significance.

## 2. Results

**Figure 1:**
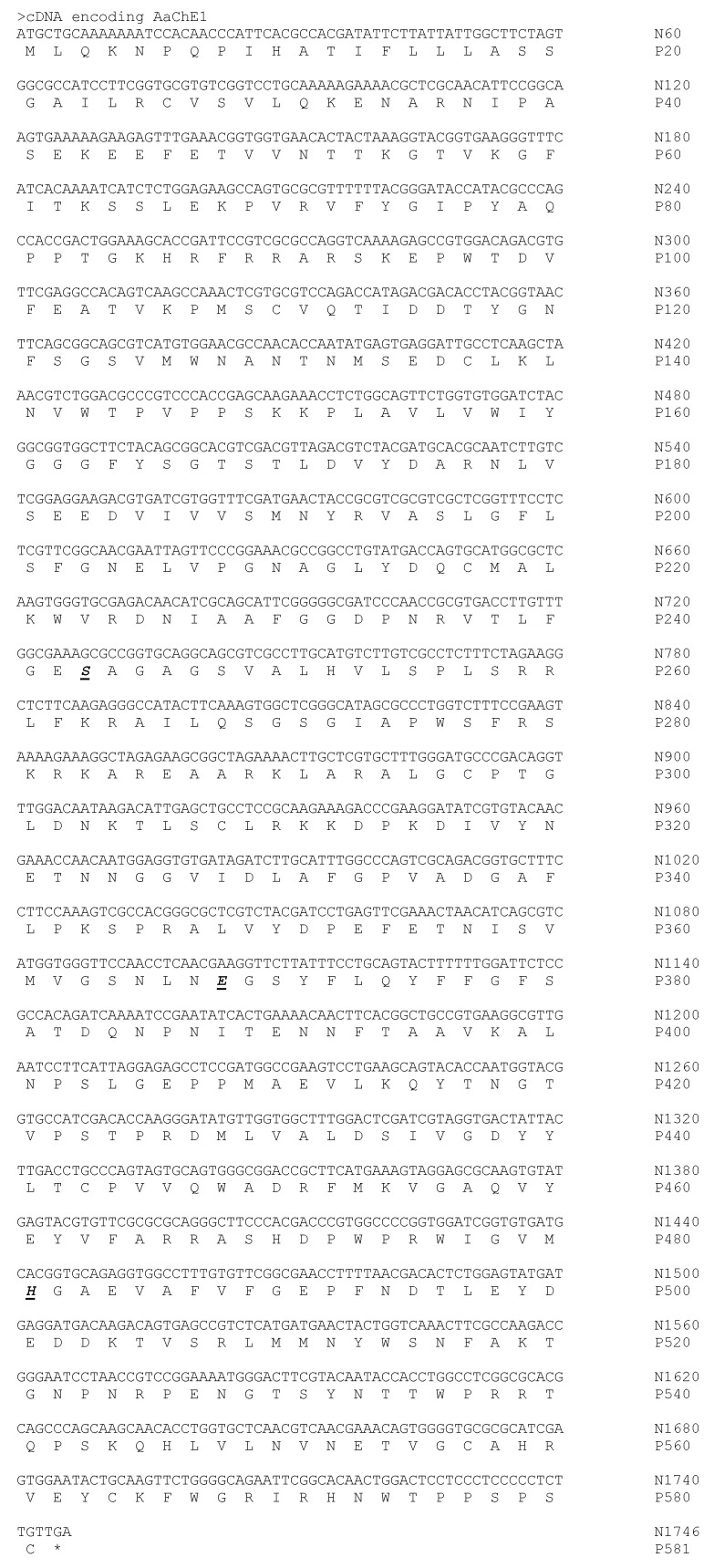
Nucleotide sequence of the *A. americanum* AChE1 cDNA (GenBank accession OQ357858) and its encoded amino acid sequence (GenBank accession WDY73567.1). Nucleotides are numbered (N#) on the right with the encoded amino acids (P#) as one letter abbreviations immediately below their codons according to the IUPAC-IUB Joint Commission on Biochemical Nomenclature. Members of the catalytic triad (S243, E368, H481) are indicated as underlined bold italics. The calculated molecular weight of the AaChE1 amino acid sequence is 64.61 kDa.

Our initial intent was to identify the salivary acetylcholinesterase of *A. americanum* [11] orthologous to BmAChE1 of the cattle tick, *Rhipicephalus microplus*. We utilized multiple sequence alignment of Acarine acetylcholinesterase mRNAs from the NCBI GenBank database to identify conserved sequence regions. We then used paired oligonucleotide primers from within the conserved sequence regions to amplify potential AChE-encoding cDNAs by reverse transcription PCR (RT-PCR) using total RNA prepared from *A. amblyomma* larvae as a template, which were subsequently sequenced and screened by a BLASTn or BLASTx search in GenBank. This process successfully identified a 938 bp cDNA tentatively identified as a partial AChE-encoding sequence, based on 917/938 (98%) nucleotide identities and 306/312 (98%) amino acid identities with NCBI accession number AJ223965.1, *R. microplus* acetylcholinesterase mRNA. Repeated primer walking by the selection of new primers (Table 1) and 5′-/3′-RACE was used to obtain the 1746 nucleotide cDNA sequence (GenBank accession OQ357858) encoding the cholinesterase, AaChE1 (581 amino acids, GenBank accession WDY73567.1) of *A. americanum* (Figure 1).

A BLASTp search of the NCBI protein database was conducted to presumptively identify the protein encoded by the complete *A. americanum* cDNA. BLAST search results revealed the highest similarity to acetylcholinesterase-like proteins of *Dermacentor andersoni* isoform X3 (accession XP_050031348.1) and X1 (accession XP_050031346.1), both exhibiting E values of 0.0 and 391/583 (67.07%) amino acid sequence identity. Analysis of conserved protein domains identified AaChE1 as a member of the carboxylesterase family (pfam00135 [16]), and analysis of the presumptive amino acid sequence of AaChE1 by Signal P 6.0 [17] indicated that there was no signal peptide, suggesting that the complete encoded amino acid sequence comprises the translated polypeptide product in vivo. Reverse transcription PCR using the AaChE1-Fwd and AaChE1-Rev primers (Table 1) and total RNA template prepared from either synganglia or salivary glands of unfed adult female *A. americanum* ticks each produced full-length cDNAs encoding amino acid sequences identical to the translated cDNA encoding AaChE1.

**Figure 2:**
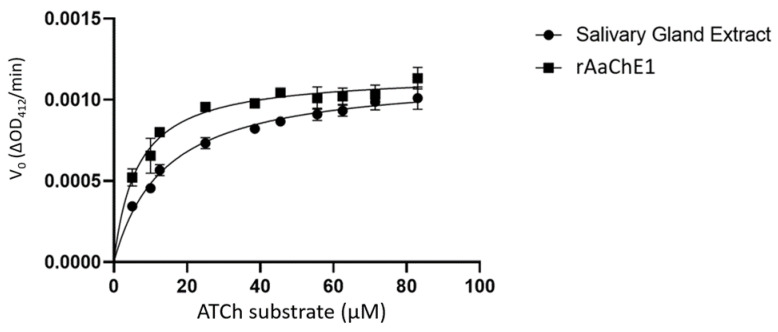
ChE activity of rAaChE1 and *A. americanum* salivary gland extract. Initial reaction velocity (V_0,_ change in optical density/min) is plotted against substrate concentration. Cholinesterase activity of rAaChE1 and salivary gland extract from *A. americanum* was measured as described in Section 4 to calculate the *K*_M_ values for acetylthiocholine as the substrate.

**Figure 3:**
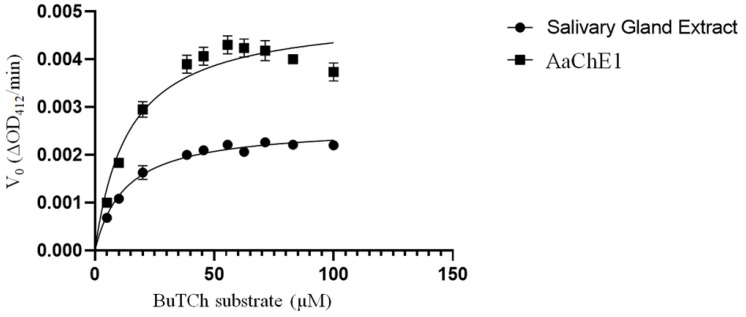
ChE activity of rAaChE1 and *A. americanum* salivary extract with butyrylthiocholine (BuTCh) substrate. Initial reaction velocity (V_0,_ change in optical density/min) is plotted against substrate concentration. Cholinesterase activity of rAaChE1 and salivary gland extract from *A. americanum* was measured as described in Materials and Methods to calculate the *K*_M_ values for BuTCh as the substrate.

**Figure 4:**
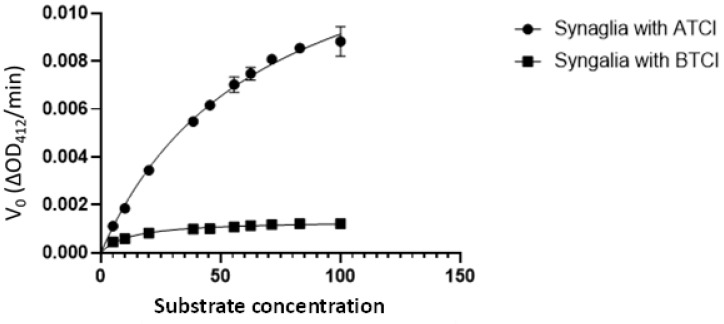
ChE activity of synganglial extract with ATCh or BuTCh as the substrates. Initial reaction velocity (V_0,_ change in optical density/min) is plotted against substrate concentration. Cholinesterase activity of *A. americanum* synganglion extract was measured as described in Section 4 to calculate the *K*_M_ values for acetylthiocholine or butyrylthiocholine as the substrate.

The coding sequence of the *A. americanum* cDNA was expressed in Sf21 insect cells and the supernatant of the baculoviral-infected cell culture was used for cholinesterase assays to determine biochemical properties of the recombinant enzyme (rAaChE1). A substrate preference assay compared acetylthiocholine (ATCh) iodide with butyrylthiocholine (BuTCh) iodide, each at 120 µM. The recombinant *A. americanum* ChE (rAaChE1) hydrolyzed BuTCh 5.74 times faster than the ATCh, demonstrating a substrate preference for BuTCh over ATCh. Salivary gland extract hydrolyzed ATCh 5.12 times faster than BuTCh, demonstrating a clear difference with rAaChE1, and providing strong evidence that AaChE1 is not the principal ChE activity present in *A. amblyomma* salivary extract. The calculated *K*_M_ (Michaelis–Menten constant) of rAaChE1 for acetylthiocholine (ATCh) was 6.39 µM (95% confidence interval of 5.17–7.81 µM). ChE activity of rAaChE1 hydrolyzing either ATCh or BuTCh was utilized to determine apparent *K*_M_ values for comparison of the total ChE activities present in salivary or synganglial extracts *of A. americanum* to that of rAaChE1. ChE assay of the salivary gland extract from 14 unfed adult female *A. americanum* ticks produced a calculated *K*_M_ for ATCh of 13.80 µM, with a 95% confidence interval of 11.91–15.99 µM (Figure 2), strongly suggesting that AaChE1 is not solely responsible for the total ChE activity present in *A. americanum* salivary gland extract. As shown in Figure 3, a comparison of rAaChE1 with salivary gland extract hydrolyzing BuTCh with apparent *K*_M_ values for rAaChE1 and salivary gland extract of 14.18 µM (95% confidence interval 10.00–19.98 µM) and 12.72 µM (95%CI, 10.66–15.16 µM), respectively, suggesting that AaChE1 may be primarily responsible for the BuChE activity present in salivary gland extract. It is notable in Figure 3 that there is apparent inhibition of rAaChE1 at BuTCh substrate concentrations of above 60 µM. Cholinesterase enzyme activity in *A. americanum* synganglial extract with either ATCh or BuTCh substrate (Figure 4) yielded apparent *K*_M_ values of 66 µM (95%CI, 56.53–77.55 µM) and 12.29 µM (95%CI, 9.61–15.68 µM) for ATCh and BuTCh, respectively, demonstrating a clear preference for the ATCh substrate in synganglial extracts of *A. americanum,* and providing strong evidence that AaChE1 is not the principle ChE present in synganglia.

The acetylcholinesterase (AChE) inhibitors eserine and BW284c51, butyrylcholinesterase (BuChE) inhibitors iso-OMPA and ethopropazine, and oxidized organophosphate acaricides, coroxon (coumaphos), malaoxon (malathion), and paraoxon (parathion), were tested to determine inhibition sensitivities of the recombinant *A. americanum* ChE (rAaChE1) hydrolyzing either ATCh or BuTCh. Inhibitor concentrations producing 50% inhibition of the rAaAChE1 (compared with the activity without inhibitor) are listed in Table 2. In general, there did not appear to be a significant difference in sensitivity to inhibitors using either ATCh or BuTCh substrates, with the exception of ethopropazine, for which 95% confidence intervals were undetermined.

**Figure 5:**
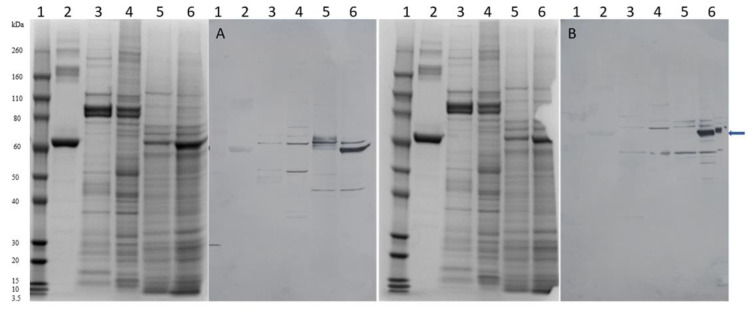
SDS-PAGE and Western blots of protein samples. **Left panel:** SDS-PAGE 4–12% PAGE gel. Samples (12 µL ea) were prepared with denaturing loading buffer and electrophoretically separated. The gel was stained with Colloidal Blue Staining kit (Invitrogen/Thermo Fisher Scientific) and blotted onto PDF membrane (**A**). Samples in lanes are: (1) Novex^TM^ Sharp Pre-stained Protein Standard (Thermo Fisher Scientific, LC5800); (2) an amount of 3.84 µg of bovine serum albumin (New England Biolabs^®^, Ipswich, MA); (3) an amount of 15.34 µg of *A. americanum* salivary gland extract; (4) an amount of 25.84 µg of *A. americanum* synganglion extract; (5) an amount of 329.9 µg of rAaChE1; (6) an amount of 328.14 µg of rBmAChE1. Molecular weights of the pre-stained protein standards (lane 1) are indicated on the left and the position of the 64.61 kDa AaChE1 is indicated by an arrow on the right. **Right panel:** SDS-PAGE 4–12% PAGE gel and blot (**B**). Samples in lanes for gel and blot as above for the left panel. **Western blots:** Blot A, 1st antibody was rabbit anti-AaChE1 (1:1000), 18 h in BLOTTO2. Blot B, 1st antibody was rabbit anti-BmAChE1 (1:1000), 18 h in BLOTTO2. After washing, each blot was incubated with mouse anti-rabbit IgG-HRP conjugate (1:5000) in BLOTTO2 for 2 h. After washing, each blot was incubated with goat anti-mouse IgG-HRP conjugate (1:5000) in BLOTTO2. After washing, horse radish peroxidase (HRP) activity developed color on reactive blot bands using diaminobenzoate/H_2_O_2_/urea (DAB/HRP stain reagent tablets, Sigma-Aldrich). Color development was stopped after 3 min.

SDS-PAGE Western blots utilizing polyclonal antibodies designed to react with either rAaChE1 or rBmAChE1 revealed that the antibodies “specific” for AaChE1 bound to PAGE-separated protein bands in extracts from salivary glands and synganglia of *A. americanum* and baculoviral supernatant expressing rAaChE1 (Figure 5), suggesting the presence of AaChE1 in synganglia and salivary extracts.

## 3. Discussion

AaChE1 is appropriately classified as a ChE (EC 3.1.1.8) based on results of BLAST searches of the NCBI databases, the enzyme activity of the recombinant protein (rAaChE1) hydrolyzing both acetylthiocholine (ATCh) and butyrylthiocholine (BuTCh), but showing a 5.74-fold preference for BuTCh compared with ATCh, relatively high sensitivity to inhibition by the acetylcholinesterase-specific inhibitors eserine and BW284c51, and relatively reduced sensitivity to inhibition by the butyrylcholinesterase-specific inhibitors iso-OMPA and ethopropazine (Table 2). The rAaAChE1 enzyme was strongly inhibited by the oxidized organophosphates, malaoxon, paraoxon, and coroxon. Identical AaChE1 amino acid sequences were encoded by cDNAs amplified from synganglia and salivary glands of adult *A. americanum* ticks suggesting expression of AaChE1 in both tissues. Comparison of total ChE activities in extracts of *A.* americanum salivary glands and synganglia revealed that unlike rAaChE1, extracts of both organs exhibited a clear substrate preference for ATCh compared with BuTCh, suggesting that if AaChE1 is present, it is likely only a minor component of total cholinesterase activity in either organ. ChE assays yielded similar BuTCh substrate-specific *K*_M_ values for rAaChE1 (14.18 µM) and salivary gland extract (12.72 µM) of *A. Amblyomma*, and the 95% confidence intervals overlapped, suggesting that AaChE1 may be the primary BuTCh-hydrolyzing component of *A. amblyomma* salivary gland extract. Similarly, BuTCh-hydrolyzing activity of *A. amblyomma* synganglial extract (*K*_M_ value, 12.29 µM BuTCh) is very similar to that of rAaChE1, suggesting that AaChE1 may be the primary BuTCh-hydrolyzing component of *A. amblyomma* synganglial extract. Cholinesterase activity in extracts of *A. amblyomma* salivary glands and synganglia both exhibit strong ATCh substrate preference and ATCh *K*_M_ values of 13.8 µM and 66 µM, respectively, compared with 6.39 µM for rAaChE1. 

Polyclonal rabbit antibodies produced in response to immunization with a peptide specific for AaChE1 bound to protein bands derived from synganglia, salivary glands, and rAaChE1 baculoviral expression supernatant in Western blots. Taken together, these data support that AaChE1 is likely expressed in synganglia and salivary glands of *A. americanum*, possibly as a component of tick saliva. Altogether, the genetic, biochemical, and immunochemical data suggest that AaChE1 is expressed in salivary glands and synganglia and may be the primary BuTCh-hydrolyzing enzyme in *A. americanum*.

Biochemical properties of the recombinant cholinesterase (rAaChE1) of *A. americanum* are different from the recombinant acetylcholinesterase 1 (rBmAChE1) of *R. (B.) microplus* [14]. However, unfed adult females of *R. (B.) microplus* and *A. americanum* each expressed cholinesterase activity in their saliva or salivary extracts [11]. The *K*_M_ values for acetylthiocholine were slightly higher in tick saliva or salivary extracts than the *K*_M_ values for acetylthiocholine obtained for the baculoviral expressed rBmAChE1 and rAaAChE1. The higher *K*_M_ values in tick saliva are likely due to the presence of other cholinesterase enzymes in addition to their respective ChE1 activities as suggested by the presence of BmAChE2 or BmAChE3-like AChEs in *R. (B.) microplus* saliva [18].

The saliva of ticks and other blood-feeding arthropod vectors have been shown to contain cholinesterase activity [11]. Acetylcholine is produced by mammalian immune and other cells, is present in mammalian tissues, and may act as a regulatory molecule to maintain homeostasis and control the development of innate and acquired immune responses [12,13,19,20,21,22,23,24,25]. The coevolution of parasites and their hosts promotes the ability of parasites to successfully manipulate or manage host immune responses, promoting the survival and reproduction of parasites [26,27]. Acetylcholine has also been reported to modulate the immune response of *Drosophila melanogaster* [28]. Arthropod vector immunity is poorly understood, including the role that the arthropod immune system has on microbe–vector interactions and its effects on arthropod populations in nature [29]. Saliva-assisted transmission (SAT) of vector pathogens is an important but poorly understood mechanism in vector-borne disease [30,31,32]. Salivary ChE injected into host tissue during blood feeding of vector arthropods is hypothesized to alter the cholinergic balance and control of developing immune responses of the host in response to tissue damage and the presence of pathogens or foreign antigens [11]. The availability of recombinant ChEs present in vector saliva will enable in vivo and in vitro studies to further elucidate the possible role(s) of salivary ChEs of arthropod vectors in host–parasite interactions.

## 4. Materials and Methods

### 4.1. Ticks, Salivary Gland Extract, Synganglion Extract, and Total RNA Preparations

A laboratory colony of *Amblyomma americanum* is maintained at the Knipling-Bushland U.S. Livestock Insects Research Laboratory and Arthropod Genomics Center (USDA-ARS, Kerrville, TX, USA) [33]. Total RNA was prepared from larvae according to the manufacturer’s instructions (Monarch^®^ Total RNA Miniprep Kit, New England BioLabs, Inc., Ipswich, MA USA; or Quick-RNA Microprep kit, Zymo Research, Irvine, CA, USA). Salivary glands or synganglia were dissected from 14 unfed adult females, placed in a DNA/RNA Shield (Zymo Research, Irvine, CA, USA ) and frozen at −80 °C until preparation of tissue specific total RNA (Monarch^®^ Total RNA Miniprep Kit, New England BioLabs, Inc., Ipswich, MA USA). Salivary gland extracts were prepared from unfed adult female ticks (without grinding) as previously described [11]. Synganglion extracts were prepared from unfed adult female ticks as described by Pruett and Pound [34].

### 4.2. RT-PCR, cDNA Construction, Cloning, and Sequencing

Total RNA from *A. americanum* larvae was amplified by RT-PCR according to the manufacturer’s instructions (Invitrogen™ SuperScript™ IV One-Step RT-PCR System, Thermo Fisher Scientific, Waltham, MA USA) using primers BmAChE1-725U19 and BmAChE1-1646L17 (Table 1). Hot-start PCR was initiated by 30 sec at 98 °C followed by 35 cycles of 10 sec at 98 °C, 10 sec at 60 °C, and 1 min at 72 °C. The cDNA amplification product was cloned, sequenced (Azenta Life Sciences, South Plainfield, NJ, USA), and searched using BLASTn and BLASTp [35,36] in the database resources of the National Center for Biotechnology Information to identify homologous sequences from the database. Gene-specific primers (Table 1) were synthesized (Sigma-Aldrich, Inc., St. Louis, MO, USA) and used to extend the cDNA sequence presumptively encoding a ChE of *A. americanum* using the SMARTer™ RACE cDNA Amplification Kit (Takara Bio, San Jose, CA, USA). After cloning and sequencing the amplification products, CLUSTAL omega [37,38] was used to align the overlapping sequences to allow the construction of a consensus cDNA sequence and gene-specific forward and reverse primers (Table 1) for RT-PCR amplification, cloning, and sequencing of the complete cDNA sequence encoding AaChE1. 

### 4.3. Baculoviral Expression of Recombinant Cholinesterases

cDNA sequences encoding complete presumptive AaChE1 constructs were cloned into pBlueBac4.5/V5-His baculoviral expression plasmid (Invitrogen, Carlsbad, CA, USA) using InFusion cloning (In-Fusion^®^ HD Cloning Kit, Takara Bio, San Jose, CA, USA) essentially as previously described [39]. PCR was initiated by 30 s at 98 °C followed by 35 cycles of 10 s at 98 °C, 10 sec at 60 °C, and 2 min at 72 °C. The expression constructs were cotransfected with Bac-N-Blue DNA (Invitrogen, Carlsbad, CA, USA) and Cellfectin reagent (Invitrogen, Carlsbad, CA, USA) according to the manufacturer’s instructions into 5 × 10^6^ *Sf*21 insect cells seeded onto 100 mm plates and overlaid with 1.5% BacPlaque agarose (Novagen, Millipore Sigma, Burlington, MA, USA) in Grace’s medium containing 150 µg/mL of Bluo-gal (5-bromo-3-indolyl β-D-galactopyranoside). Blue plaques were picked and used to infect 25–50% confluent Sf21 cells in 5 mL of Sf900 III SFM insect medium (Thermo Fisher Scientific, Waltham, MA USA). AChE activity of culture supernatant was monitored daily beginning 2 d after picking plaques and continuing until ChE activity reached a maximum or complete cell death in the plaque culture flasks. Baculoviral-expressed recombinant AaChE1 preparations were harvested as baculoviral culture supernatants.

### 4.4. Cholinesterase Assays

Cholinesterase assays were performed as described previously [14], including substrate preference, determination of *K*_M_ values, and sensitivity to inhibitors. All enzyme assays utilized the baculoviral supernatant of *Sf*21 cell cultures not expressing rAaChE1 as a negative control. For inhibition assays, the enzymes were preincubated with inhibitor for 15 min (unless otherwise specified) prior to initiation of the reaction by the addition of the substrate. Each assay utilized triple replicate wells in the same bioassay plate. Progress of the reaction was monitored at 412 nm using a Bio-Tek EL808 Ultra Microplate Reader (Bio-Tek Instruments, Inc., Winooski, VT, USA) with readings every min for a total of 30 min. The negative control (substrate only, no active enzyme) was used to determine the background, which was subtracted from the absorbance values of equivalent time reactions to yield reaction absorbance values over time. Only linear reaction rates were used to determine the initial velocities (V_0_) at each test condition, which were utilized to determine kinetic parameters. Kinetic parameters for rAaChE1 assays and inhibitor concentrations producing 50% reduction (IC_50_) in enzyme activity without inhibitor were determined by probit regression analysis in GraphPad Prism ver. 5.0 (GraphPad Prism, Inc., La Jolla, CA, USA). Confidence intervals of 95% (95% CI) for K_M_ and IC_50_ values were calculated by GraphPad Prism. Statistical significance (*p* ≤ 0.05) was determined with GraphPad Prism using ANOVA and the least significant difference t-test for the comparison of means.

### 4.5. AaChE-Specific Antibody and SDS-PAGE Western Blot

Polyclonal rabbit antibodies specific for rAaChE1 or rBmAChE1 (recombinant acetylcholinesterase 1 of the cattle tick, *R. [B.] microplus*) [14] were designed and prepared by GeneScript Biotech Corporation (Piscataway, NJ, USA). Rabbits were immunized with peptide-KLH conjugate (rAaChE peptide, CAKTGNPNRPENGTS; rBmAChE1 peptide, TGKRRFDRAESIEEC). SDS-PAGE gels (Bold 4–12% Bis-Tris Plus (NW04122BOX), Thermo-Fisher Scientific) and Western blot onto PVDF membranes were conducted as previously described [40] with 12 µL of sample loaded into each well. Blots were blocked by 30 min of incubation in 10% dried goat milk (Hoosier Hill Farm, Fort Wayne, IN, USA), incubated overnight (18 h) with the first antibody (rabbit anti-BmAChE1 or rabbit anti-AaChE1) diluted 1:1000 in BLOTTO2 (2% goat milk, 1% Tween-80), washed 5 times (5 min each) in wash buffer (0.01 M NaPhosphate, pH 7.4, 0.3% Tween-20), incubated 2 h with 2nd antibody (mouse anti-rabbit IgG-HRP conjugate, Sigma Chemical, St. Louis, MO, USA) diluted 1:5000 in BLOTTO2, washed 3 times as before, incubated 30 min with 3rd antibody (goat anti-mouse IgG-HRP conjugate, Bio-Rad Laboratories, Inc., Hercules, CA, USA) diluted 1:5000 in BLOTTO2, washed as previously described, and then color developed for 3 min in DAB/H_2_O_2_ urea (Sigma Chemical, ST. Louis, MO, USA).

## Figures and Tables

**Table 1:** Oligodeoxynucleotide primers.

Name	Sequence (5′-3′)	Purpose
BmAChE1-725U19	TGGAAATGCCGGACTATAC	RT-PCR *A. americanum* larval RNA
BmAChE1-1646L17	CGGTCTTGGCGAAGTTG	RT-PCR *A. americanum* larval RNA
AaChE573U18	CGGCGGTGGCTTCTACAG	3′-RACE *A. americanum* larval RNA
AaChE186L18	CTTGAGCGCCATGCACTG	5′-RACE *A. americanum* larval RNA
AaChE-Fwd	AGAATTGCCCTTATGCTGCAAAAAATCCACACAAG	Clone into TOPO XL-2 plasmid
AaChE-Rvs	CGAATTGCCCTTTCAACAAGAGGGGGAGGGAG	Clone into TOPO XL-2 plasmid
pBB-BmAChE1-AaFwd	TGAAAGGGCAATTCGAAGC	InFusion clone into baculoviral expression plasmid
pBB-BmAChE1-AaRvs	CATAAGGGCAATTCTAGACC	InFusion clone into baculoviral expression plasmid

**Table 2:** IC50 values for rAaChE1 with acetylthiocholine (ATCh) or butyrylthiocholine (BuTCh) substrate.

Inhibitor ^1^	(IC50) ^2^	
	rAaChE1 (ATCh Substrate)	rAaChE1 (BuTCh Substrate)
Eserine	2.28 × 10^−10^ (2.37–3.26 × 10^−10^)	9.06 × 10^−10^ (5.46–14.96 × 10^−10^)
Iso-OMPA	3.48 × 10^−05^ (2.76–4.43 × 10^−5^)	2.77 × 10^−5^ (2.42–3.17 × 10^−5^)
Ethopropazine	6.82 × 10^−5^ (N.D.)	2.27 × 10^−04^ (N.D.)
BW284c51	2.96 × 10^−7^ (2.50–3.50 × 10^−7^)	1.46 × 10^−7^ (1.29–1.64 × 10^−7^)
Coroxon	2.35 × 10^−11^ (1.93–2.84 × 10^−11^)	9.07 × 10^−12^ (0.91–2.26 × 10^−11^)
Malaoxon	2.77 × 10^−8^ (2.45–3.14 × 10^−8^)	3.59 × 10^−08^ (1.40–1.93 × 10^−8^)
Paraoxon	1.31 × 10^−9^ (1.13–1.52 × 10^−9^)	1.08 × 10^−9^ (0.92–1.27 × 10^−9^)

^1^ AChE-specific inhibitor BW284c51 (1,5-Bis(4-allyldimethylammoniumphenyl)pentan-3-one dibromide, Sigma); BuChE-specific inhibitors iso-OMPA (tetraisopropylpyrophosphoramide, Sigma) and ethopropazine hydrochloride (Sigma). ^2^ Concentration of inhibitor producing 50% inhibition; N.D., not determined.

## Data Availability

Messenger RNA sequences (GenBank accession OQ357858) encoding AaChE1 and the encoded amino acid sequences of AaChE1 (GenBank accession WDY73567.1) are archived in databases at the National Center for Biotechnology Information (NCBI).

## Abbreviations

AaChE1, *Amblyomma americanum* cholinesterase 1; AChE, acetylcholinesterase; alpha-gal, galactose-alpha-1,3-galactose; ATCh, acetylthiocholine; BLAST, Basic Local Alignment Search Tool; BLASTn, nucleotide BLAST; BLASTp, protein (or peptide) BLAST; BLASTx, translated nucleotide BLAST; BmAChE1, *Rhipicephalus microplus* acetylcholinesterase 1; BuChE, butyrylcholinesterase; BuTCh, butyrylthiocholine; BW284c51, 1,5-Bis(4-allyldimethylammoniumphenyl)pentan-3-one dibromide; cDNA, complimentary DNA; ChE, cholinesterase; CLUSTAL, computer software used for multiple sequence alignment; DNA, deoxyribonucleic acid; EC 3.1.1.8, Enzyme Commission number identifying cholinesterases; HRP, horse radish peroxidase; Iso-OMPA, tetraisopropyl pyrophosphoramide; *K*_M_, Michaelis–Menten constant; mRNA, messenger RNA; NCBI, National Center for Biotechnology Information; PCR, polymerase chain reaction; PCR, polymerase chain reaction; PVDF membrane, polyvinylidene difluoride membrane; rAaChE1, recombinant AaChE1; rBmAChE1, recombinant BmAChE1; RNA, ribonucleic acid; RT-PCR, reverse transcriptase polymerase chain reaction; SDS, sodium dodecyl sulfate; *Sf*21, an insect cell line derived from *Spodoptera frugiperda*; TOPO cloning, topoisomerase I-activated cloning; and V_0_, initial reaction velocity.
